# Visual-Spatial and Verbal Remote Association: An fMRI Study

**DOI:** 10.3389/fpsyg.2021.672997

**Published:** 2021-08-10

**Authors:** Ching-Lin Wu, Hsueh-Chih Chen

**Affiliations:** ^1^Program of Learning Sciences, National Taiwan Normal University, Taipei, Taiwan; ^2^Institute for Research Excellence in Learning Sciences, National Taiwan Normal University, Taipei, Taiwan; ^3^Department of Educational Psychology and Counseling, National Taiwan Normal University, Taipei, Taiwan; ^4^Chinese Language and Technology Center, National Taiwan Normal University, Taipei, Taiwan

**Keywords:** remote association, creativity, visual-spatial, verbal, fMRI

## Abstract

Although idea connections at verbal and conceptual levels have been explored by remote associates tests, the visual-spatial level is much less researched. This study investigated the visual-spatial ability via Chinese Radical Remote Associates Test (CRRAT), wherein respondents consider the positions of the stimulus and target Chinese radicals. Chinese Compound Remote Associates Test (CCRAT) questions also feature stimuli of a single Chinese character; therefore, it was adopted for comparison to distinguish the roles played by verbal and visual-spatial associations in a remote associative process. Thirty-six adults responded to CRRAT and CCRAT; their brain activities were analyzed. Upon excluding the influence of age, verbal comprehension, and working memory, it was found that the caudate, posterior cingulate cortex, postcentral gyrus, and medial frontal gyrus were activated when the respondents answered CCRAT, but only the caudate showed significant activation when they answered CRRAT. The Chinese radical remote association minus the Chinese compound remote association showed that the middle frontal gyrus, inferior parietal lobule, and precuneus demonstrated significant activation. Therefore, this study demonstrated differences in brain mechanisms between visual-spatial and verbal remote associations.

## Introduction

Based on the associative theory of creativity, remote association refers to the ability of one to form new relations with seemingly irrelevant elements (Mednick, 1962). Those with high remote associative abilities are considered to be able to produce unusual and novel ideas that represent creativity (Wu et al., 2017; Wu, 2019). Remote associative ability is often evaluated using the remote associates test (RAT) (Mednick, 1968). A RAT question includes three remotely associated English stimuli, and respondents are asked to propose an English word that can pair up with each of the stimuli to create three meaningful expressions. For instance, in a RAT question that consists of three stimuli, “blood,” “music,” and “cheese,” and one possible solution, “blue,” the target word can be combined with the stimuli to create meaningful expressions such as “blue blood,” “blue music,” and “blue cheese,” respectively.

In addition, RAT has a short testing duration and objective scoring. Furthermore, its questions are relatively easy to compile, which facilitates the mass production of RAT questions to prevent respondents from knowing the test questions beforehand. Consequently, RAT has been widely used in creativity research on different dimensions (Wu et al., 2020). Moreover, it has been translated into diverse versions in different languages, such as Dutch (Akbari et al., 2012), Japanese (Terai et al., 2013; Orita et al., 2018), Italian (Salvi et al., 2016), and Chinese (Shen et al., 2016; Xiao et al., 2016; Wu and Chen, 2017).

Chinese RATs (i.e., CRATs) have three versions: the Chinese Radical Remote Associates Test (CRRAT) (Wu and Chen, 2017), the Chinese Compound Remote Associates Test (CCRAT) (Chang Y. L. et al., 2016), and the Chinese Word Remote Associates Test (CWRAT) (Huang et al., 2012). They are at three different levels of the Chinese language, namely Chinese radicals, characters, and words. Among these three versions, CRRAT questions involve relative positions of Chinese radicals, while stimuli and target Chinese radicals involved in a CRRAT question often have two spatial relationships that are horizontal (two involved Chinese radicals of a Chinese character, with one on the left and the other on the right) and vertical (two involved Chinese radicals, with one at the top and the other at the bottom). Contrary to typical verbal associations, CRRAT questions also test visual-spatial associative abilities. Empirical studies have found that CCRAT and CRRAT are positively correlated with verbal and visual divergent thinking, respectively (Wu, 2019), indicating that CRRAT may involve visual-spatial associations. The findings reveal more ways by which participants form associations when they respond to different CRATs.

Research on the brain mechanism associated with creativity has revealed a wealth of findings through interdisciplinary studies and up-to-date equipment (Cerruti and Schlaug, 2009; Brunyé et al., 2015; Wu C.-L. et al., 2016; Aberg et al., 2017; Bendetowicz et al., 2018; Pick and Lavidor, 2019; Wu et al., 2019). Based on these behavioral findings, this study searched for relevant physiological evidence. To achieve this goal, this study compared the brain regions that were activated when respondents formed verbal and visual-spatial remote associations, and distinguished between the corresponding physiological mechanisms by comparing and contrasting the brain activities that they undertook when responding to CCRAT and CRRAT questions.

## Associations Other Than Verbal in Chinese Remote Associates Test

In Chinese-speaking areas, three versions of CRAT were developed based on Chinese radicals, characters, and words, which are the three levels of the Chinese language. The question-answer process for CRATs may involve different remote associations (Wu, 2019).

CRRAT was developed by Chang Y. L. et al. (2016) based on the manner by which a typical RAT was compiled. The CRRAT questions consist of three Chinese radicals as stimuli, requiring respondents to propose a target Chinese radical that can pair with all the stimuli to form meaningful Chinese characters. For instance, a CRRAT question has three stimuli “女” (nü; female), “子” (tzu; son), and “禾” (ho; standing grain), and the Chinese radical “乃” (nai; be) is one possible answer, which can be combined with the stimuli to form “奶” (nai; milk; the stimulus Chinese radical “女” on the left with the target Chinese radical “乃” on the right), “孕” (yün; pregnancy; the target Chinese radical “乃” at the top with the stimulus Chinese radical “子” at the bottom), and “秀” (hsiu; elegance; the stimulus Chinese radical “禾” at the top with the target Chinese radical “乃” at the bottom). The Chinese radicals used as stimuli in this study were selected from the Chinese Orthography Database (Chen et al., 2011). The above example shows two ways that Chinese radicals are combined to form a Chinese character, horizontal (such as “奶”) and vertical (such as “孕”). As another example, the Chinese character “明” involves a horizontal combination of the radicals “日” and “月,” i.e., 明=日月, whereas the Chinese character “貢” is formed *via* the vertical combination of the Chinese radicals “工” and “貝,” i.e., 貢=工貝. Therefore, CRRAT participants will not find correct answers to the questions until they consider the relative position of the stimuli and target Chinese radicals.

In addition, the CCRAT was developed based on the CRAT that was first used (Jen et al., 2004). CRAT questions consist of three Chinese characters, such as “今” (chin; now), “輕” (ching; light), and “去” (chu; go), requiring participants to propose a target Chinese character that can be paired with all the three single-character Chinese stimuli to form meaningful two-character Chinese words. For the above example, “年” (nien; year) is a possible solution, which can be paired with the stimuli to create the Chinese words “今年” (chin-nien; this year), “年輕” (nien-ching; being young), and “去年” (chu-nien; last year), respectively. Later, Wu et al. (2017) set the usage frequency of Chinese words formed based on the stimuli and target word within the last one-third (the least used) when compiling CRAT questions. It was found that CRAT performances of participants were positively correlated with divergent thinking and insight problem-solving, indicating that its criterion-related validity can be improved if it is compiled in this way. Consequently, Wu and Chen (2017) normalized the data, such as the percentage of correct answers and the time that it took respondents to give correct responses, and renamed the CRAT that manipulated the usage frequency as CCRAT to distinguish between the two.

Finally, CWRAT was compiled by Huang et al. (2012) based on the three ways by which remote associations are formed, namely synonymy, formation of a compound word, and semantic association, which were proposed by Mednick (1968) and Bowden and Jung-Beeman (2003). The CWRAT questions consist of three Chinese words, asking participants to think of a two- or three-character Chinese word that can be combined with the stimuli to create “relevant” meaningful Chinese words; here, “relevant” refers to a semantic association or the ability to form four-character Chinese compound words. For instance, for a CWRAT question consisting of three stimuli “市場” (shih-chang; market), “結束” (chieh-shu; an end), and “夕陽” (hsi-yang; sunset), and one possible solution “黃昏” (huang-hun; dusk), the Chinese characters “黃昏” (huang-hun; dusk) can be paired with “市場” (shih-chang; market) to form the four-character Chinese compound word “黃昏市場” (huang-hun shih-chang; dusk market), which is semantically associated with “結束” (chieh-shu; an end), for dusk signifies the end of the day and is synonymous to “夕陽” (hsi-yang; sunset), because sunset occurs at dusk. The value of criterion-related validity of CWRAT to insight-problem solving fell between 0.4 and 0.51, indicating that the question-answer process of CWRAT is most similar to that of insight-problem solving among the three CRATs.

Overall, CRATs at different levels (Chinese radicals, characters, and words) consist of disparate test questions, for which respondents use different language knowledge and strategies during the question-answer process. When responding to CCRAT questions, research participants need to think of remotely associated concepts based on the stimuli in order to find out correct answers, because the usage frequency of the created words is set as “the least used” (Wu et al., 2017). Empirical studies have found that Chinese compound remote association is positively correlated with the fluency, flexibility, and originality of verbal divergent thinking (Wu, 2019), indicating that CCRAT involves multiple levels of verbal divergent thinking.

Moreover, contrary to the alphabetic system, Chinese characters have complex spatial properties; therefore, it is necessary to perform visual-spatial processing during Chinese orthography (Lu et al., 2011; Chang L.-Y. et al., 2016; Yang et al., 2018). Therefore, individuals need to consider the relative positions of Chinese radicals when responding to CRRAT (Taft et al., 2000), which involves visual-spatial abilities. For instance, to solve the above test question that consists of “女” (nü; female), “子” (tzu; son), and “禾” (ho; standing grain), respondents first need to search for the Chinese radicals that can form meaningful Chinese characters with the stimuli, such as “子” (女子, 好; hao, good), “乃” (女乃, 奶; nai, milk), “系” (子系, 孫 sun, grandchild), “乃”乃子, 孕; yün, pregnancy), and “乃” (禾乃秀; hsiu, excellence). They also need to consider the relative positions of the stimulus and target Chinese radicals; for instance, to form the Chinese character “奶” (nai, milk), the stimulus radical “女” is placed on the left with the target “乃” on the right; to create the Chinese character “秀” (hsiu, excellence), the stimulus radical “禾” is placed at the top with the target “乃” at the bottom. Behavioral research has demonstrated that CRRAT is positively associated with the originality of visual divergent thinking (Wu, 2019). Furthermore, another empirical study also found that CRRAT is positively correlated with the connectivity of the temporoparietal junction and the posterior inferior parietal lobule (Wu and Chen, 2021), which involves visual attention and visual-spatial information processing (Pisapia et al., 2016; Pedrazzini et al., 2017). In brief, Chinese radical remote association may involve visual-spatial ability based on the complex spatial properties of Chinese characters.

Lastly, when research participants respond to CWRAT, they often answer questions based on two-character Chinese words, finding possible answers through semantic meaning or semantic association of stimulus Chinese words and the way Chinese words are combined. Previous studies have revealed that CWRAT performance is positively correlated with that of insight-problem solving (Huang et al., 2012; Wu, 2019), suggesting that the internal process that one goes through when responding to CWRAT differs from those for CCRAT and CRRAT.

To summarize, CCRAT and CRRAT both require participants to think of a Chinese character, while the former requires them to combine the stimulus with the target to form a two-character Chinese word, and the latter requires them to create a totally new Chinese character. On the contrary, CRRAT respondents need to consider the relative position between the stimulus and the target, for which they need to use their visual-spatial ability when searching for answers (Chang Y. L. et al., 2016), while CCRAT does not involve this ability. In this respect, CCRAT and CRRAT can be used to explore verbal and visual-spatial remote associations, respectively, and the former can also be used for comparison.

## Research Findings of Remote Association and Brains

In recent years, a growing number of studies have explored the relationship between remote associations and human brains in the context of cognitive neuroscience (Wu et al., 2020). The pre-frontal and parietal lobes have been found to be correlated with remote associations (Bendetowicz et al., 2017, 2018). Based on the topological properties of brain networks, the connection efficiency between the nodes of the middle temporal gyrus, inferior parietal lobule, insula, median cingulate, fusiform gyrus, angular gyrus, calcarine fissure, and superior parietal gyrus is positively correlated with remote associative performance (Wu C.-L. et al., 2016).

The RAT problem-solving process includes two stages: idea production (divergent thinking) and idea assessment (convergent thinking) (Smith et al., 2013). Divergent thinking is associated with the ability of individuals to fluently produce novel ideas of different types, which involve the brain activity of the frontal lobe, temporal lobe, caudate, and posterior cingulate cortex (Benedek et al., 2014a,b, 2016; Cousijn et al., 2014; Yoruk and Runco, 2014; Jauk et al., 2015; Wu C.-L. et al., 2016; Kleibeuker et al., 2017; Sunavsky and Poppenk, 2020; Wertz et al., 2020). On the contrary, convergent thinking is associated with the ability to categorize and collate produced ideas and evaluate the feasibility and validity of the ideas, which facilitates finding the most appropriate answer. It involves the brain activity of the frontal lobe, temporal lobe, precuneus, and amygdala (Shen et al., 2018; Wu et al., 2019).

In addition, visual creativity refers to how one uses spatial imagery to produce visualized innovative products (Pisapia et al., 2016). Previous research has found that when drawing, designing, or evaluating creative products during image creativity tests, the following brain regions present significant activation: thalamus, fusiform gyrus, middle and inferior frontal gyri, and superior and inferior parietal lobules (Yomogida et al., 2004; Ellamil et al., 2012; Aziz-Zadeh et al., 2013; Huang et al., 2013; Park et al., 2015; Saggar et al., 2015; Pidgeon et al., 2016; Tian et al., 2018).

In conclusion, diverse types of creative problem-solving are associated with the activity of different brain regions (Wu et al., 2020), such as the frontal lobe, temporal lobe, parietal lobe, posterior cingulate cortex, precuneus, and caudate (Jauk et al., 2015; Shen et al., 2018; Wu et al., 2019; Wertz et al., 2020). These findings suggest that brain activity can reflect the performance of creative thinking. Therefore, the brain mechanisms of visual-spatial and verbal associations may also be closely associated with these regions, so this requires further confirmation.

## This Study

Typical RATs are often used to assess the ability of one to form lexical or conceptual associations of ideas (Wu and Chen, 2017; Toivainen et al., 2019); they are rarely employed to test visual-spatial associations. Therefore, this study compared CCRAT and CRRAT, both of which adopt single Chinese characters (Chinese radicals are often Chinese characters as well) as stimuli to distinguish the roles of verbal and visual-spatial associations in the problem-solving process of CRATs.

This study aimed to differentiate the attributes of the remote associations involved in CCRAT and CRRAT. Previous studies have indicated that verbal intelligence, working memory, and remote association are potentially correlated; that is, they are not always significantly correlated (Lee and Therriault, 2013; Lee et al., 2014; Wu C.-L. et al., 2016). This research collected data on not only the two versions of CRAT performance but also verbal comprehension and working memory. After excluding the influence of verbal comprehension and working memory, we compared the correct rates and brain activation involved in the two types of Chinese remote associations. These findings provide further empirical evidence on the cognitive process of remote associations by distinguishing the neural mechanisms for verbal and visual-spatial associations.

To summarize, the pre-frontal and parietal lobes are associated with remote associations (Bendetowicz et al., 2017, 2018). The following brain regions have been found to be associated with divergent thinking: frontal lobe, temporal lobe, caudate, and posterior cingulate cortex (Benedek et al., 2014a,b, 2016; Cousijn et al., 2014; Jauk et al., 2015; Wertz et al., 2020). In addition, the frontal lobe, temporal lobe, precuneus, and amygdala are all associated with convergent thinking (Shen et al., 2018; Wu et al., 2019) and play different roles in remote association. Moreover, the middle and inferior frontal gyri, as well as superior and inferior parietal lobules, are all associated with visual creativity (Ellamil et al., 2012; Aziz-Zadeh et al., 2013; Huang et al., 2013; Park et al., 2015; Saggar et al., 2015; Pidgeon et al., 2016). Based on these findings, this research assumes that verbal association is associated with the activation of the brain regions of the temporal lobe, precuneus, and posterior cingulate cortex, while visual-spatial association is associated with the activation of the brain regions of the middle and inferior frontal gyri and the superior and inferior parietal lobules.

## Materials and Methods

### Participants

A total of 60 adults (24 men and 36 women) voluntarily participated in the study. Their ages fell between 20 and 30 years, with a mean of 24.1 ± 2.65 years. All of the participants were university students or had a university degree. The Edinburgh Handedness Inventory (Oldfield, 1971) was used to ensure that all the participants were right-handed. In addition, their native language is traditional Chinese, and there is no obvious difficulty in reading it. Moreover, it was confirmed that they did not consume any alcoholic drinks 24 h before the experiment. Furthermore, this experiment was approved by the Institution Review Board of the National Taiwan Normal University, and all the participants had fully understood the research and signed an informed consent form before it was conducted.

To effectively analyze the differences in the conditions of brain images, this research only kept the data of the respondents who had no <10 correct and 10 unsolved answers for CCRAT, which are represented as CCRAT correct (CC) and CCRAT unsolved (CU), respectively, and who had no <10 correct and 10 unsolved responses for CRRAT, which are denoted as CRRAT correct (RC) and CRRAT unsolved (RU), respectively. The brain images of 36 respondents were reserved for subsequent analysis, of which 13 were male and 23 were female, with a mean age of 23.88 ± 2.56 years.

### Measures

This study used CCRAT, CRRAT, and Wechsler Adult Intelligence Scale-IV (WAIS-IV) as research tools, as discussed below.

#### Chinese Compound Remote Associates Test (CCRAT)

The CCRAT employed in this study was compiled by Wu et al. (2017). It consists of 30 questions, with each question composed of three stimuli that are single Chinese characters, such as “療” (liao; treatment), “防” (fang; defense), and “統” (tung; completely). The participants were asked to propose a Chinese character that can be combined with all three stimuli to create three meaningful two-character Chinese words. For this example, a possible solution for this question is “治” (chih; ruling), as it can be combined to form the two-character Chinese words “治療” (chih-liao; treatment), “防治” (fang-chih; prevention), and “統治” (tung-chih; ruling), respectively. The participants were given a point for every correct answer. The higher the score, the better the remote associative ability. Their performance was represented by the pass rate (i.e., the percentage of correct answers) in this study.

#### Chinese Radical Remote Associates Test (CRRAT)

In this study, CRRAT, compiled by Chang Y. L. et al. (2016), was used. It consists of 30 questions. Each test question comprised of three Chinese radicals, e.g., “女” (nü; female), “子” (tzu; son), and “禾” (ho; standing grain). The participants were required to propose a Chinese radical that can be combined with these three stimuli to form meaningful and commonly used Chinese characters. For this example, “乃” (nai; be) is one possible answer. The participants were given a point for every correct answer. The higher the score, the better the remote associative ability.

#### Wechsler Adult Intelligence Scale-IV (WAIS-IV)

Chen et al. (2015) translated the WAIS-IV used in this research from English into Chinese. The Chinese WAIS-IV is applicable to those aged no <16 years but no more than 100 years. The respondents were scored in four categories: verbal comprehension, perceptual reasoning, working memory, and processing speed. They were also scored in terms of four selectivity indexes: general ability, cognitive proficiency, visual-spatial, and fluid reasoning. The WAIS-IV consists of 15 subtests: similarities, block design, digit span, symbol search, vocabulary, matrix reasoning, letter-number sequencing, coding, information, visual puzzles, arithmetic, cancelation, comprehension, figure weights, and picture completion. The split-half reliability fell between 0.91 and 0.98, while the test-retest reliability was between 0.86 and 0.97. WAIS-III was employed as the criterion task, with concurrent validity falling between 0.78 and 0.95. Normative data for Taiwan were used as a reference for this test. RAT is thought to be greatly influenced by the verbal competence of participants. Consequently, this study only implemented the verbal comprehension and working memory subscales of WAIS-IV.

### Experimental Paradigm

This study adopted an event-related functional magnetic resonance imaging paradigm. The gazing point “+” was presented the moment a stimulus appeared on the screen, which lasted 6 s (5.3, 5.65, 6.1, 6.25, and 6.7 s) on average. Subsequently, a test question was presented on the screen for, at most, 20 s, which included three stimuli of Chinese characters. If the participants had answers to questions within the time limit, they could press the button and move on to question-answering screens, reporting their answer orally in 5 s. To determine whether the answers were correct, their answers were noted on the answer sheet. After finishing the check, the examiner presented the next question manually, asking the participants to continue answering questions. If the participants did not think of any answer within 20 s, the screen for the participants to report answers would appear automatically, but the examiner would not allow the participants to report their answers orally; the examiner would then manually move on to the next question and ask them to answer it. In summary, the stimuli onset asynchrony (SOA) was 25.3, 25.65, 26.1, 26.25, and 26.7, respectively. Each block consisted of 15 questions, which took up to 465 s. There were four intervals in total, with a 2-min break between each interval. The formal experiment took up to 37 min. The exact time relied on the actual question-answering situation.

### Image Acquisition

Magnetic resonance imaging (MRI) scan was conducted using a Siemens high-field magnetic resonance scanner (Siemens, Munich, Germany) equipped with a 64-channel head coil with a 90-degree phase difference and input/output (I/O) functions at the Research Center for Mind, Brain, and Learning of the National Chengchi University. Stimuli were presented with E-prime 3.0, and respondents read the stimuli that were projected on a projection screen via a mirror in front of them. The axial view was utilized to scan the whole brain with the anterior commissure-posterior commissure (AC-PC) line as the imaging reference line. Echo-planar imaging (EPI) was performed with the following parameters: TR = 2,000 ms, TE = 25 ms, FA = 90°, matrix size = 224 × 224 mm^2^, FOV = 224 × 224 mm^2^, slice number = 40, thickness = 3 mm, and spatial resolution = 3.5 × 3.5 × 3 mm^3^.

Subsequently, a high-resolution T1-weighted anatomical image scan, which was employed to digitize activated and functionally connected brain regions, was conducted. The pulse sequence of the anatomical image was 3D-MPRAGE, and the related parameters were as follows: TR, 2,530 ms; TE, 3.3 ms; FA, 7°; matrix size, 256 × 256 mm^2^; FOV, 256 × 256 mm^2^; slice number 192; thickness, 1 mm; and spatial resolution, 1 × 1 × 1 mm^3^.

### Image Analysis

The collected data were analyzed using SPM12 (Statistical Parametric Mapping, Wellcome Department of Cognitive Neurology, London, United Kingdom). Functional images were corrected for differences in slice acquisition time to the middle volume, and they were realigned to the first volume in the scanning session using affine transformations. The movement in any plane did not exceed 4 mm. Co-registered images were normalized to the standard Montreal Neurological Institute EPI template and resampled to a voxel size of 3 × 3 × 3 mm. The statistical analyses were performed on data that had been spatially smoothed using an 8-mm (FWHM) Gaussian kernel, with a high-pass filter (with a cutoff period of 128 s) at full width at half maximum to remove low-frequency artifacts.

For the event-related analysis, functions corresponding to the onset of different event types were constructed and convolved with a canonical hemodynamic response function (HRF) and its temporal derivative. During the first-level single-subject analysis, different types of events (CC, CU, RC, and RU) were defined, and voxel-wise parameter estimation was calculated for each regressor. In this study, “unsolved” implied that the participants did not submit any response during the trial. Canonical HRF was used to analyze stimuli as separate events, which were divided into solved and unsolved (2 s before pressing the button or the end of the response time). To increase the statistical sensitivity and remove motion-related artifacts, we also included six motion parameters as regressor/nuisance covariates of no interest in the first-level general linear model.

The individual contrast images were then entered into a second-level analysis using a flexible factorial design. Parametric modulation was analyzed by paired *t*-tests, which allowed the study to parse its main effects on solving the two types of remote association (CCRA/CRRA). Meanwhile, the difference in correct events between the Chinese Compound and Radical Remote Associates problem solving was also analyzed by paired *t*-tests.

All reported areas of activation were considered significant at the peak threshold of *p* < 0.05, corrected for the family-wise error rate across the whole brain for multiple comparisons at the voxel level, with a cluster size ≥20 voxels. To visualize significant signal changes in the brain regions, time courses were extracted from the beta values for the peak voxel.

## Results

### Behavioral Results

Table 1 presents the means of the correct answer rate, standard deviations (SD), and correlation coefficients for verbal comprehension, working memory, CCRAT, and CRRAT. Regarding the mean correct rate, CCRAT was 0.42 (*SD* = 0.07) while CRRAT was 0.48 (*SD* = 0.08). In terms of verbal intelligence, the mean verbal comprehension was up to 122.22 ± 8.31, while that of working memory was up to 110 ± 14.08. The demographic variables (gender, age, verbal comprehension, and working memory) were not significantly correlated with Chinese compound remote association and Chinese radical remote association (*rs* < 0.28, *ps* >0.1).

**Table 1:** Descriptive statistics and correlation coefficients of variables.

	**Descriptive statistics**		**Correlations**					
	**Mean**	**S.D**.	**Gender**	**Age**	**WM**	**VC**	**CCRAT**	**CRRAT**
Age	23.88	2.56	−0.16	–				
WM	110.00	14.08	0.004	0.03	–			
VC	122.22	8.31	−0.21	−0.09	0.43^**^	–		
CCRAT	0.42	0.07	0.04	−0.25	−0.01	0.04	–	
CRRAT	0.48	0.08	0.28	0.02	−0.01	−0.11	0.16	–

*WM, working memory; VC, verbal comprehension; CCRAT, Chinese Compound Remote Associates Test; CRRAT, Chinese Radical Remote Associates Test*.

** *p < 0.01*.

### fMRI Results

#### Different Types of Remote Association

**Figure 1:**
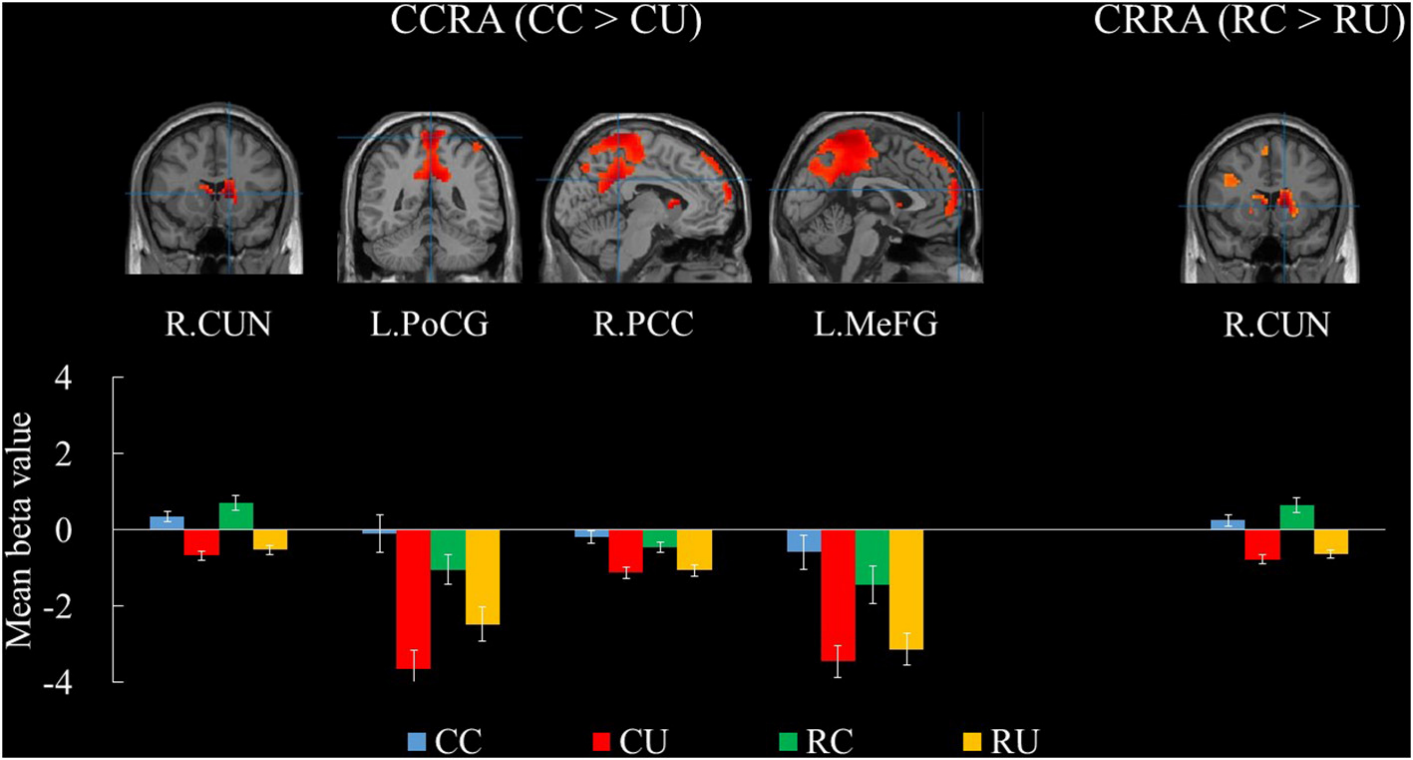
Brain regions for Chinese Compound or Radical Remote Associations. The bars show the mean beta values of the peak voxels for each of the four conditions. Error bars represent standard error of the mean. L, left; R, right; CUN, caudate; PoCG, postcentral gyrus; PCC, posterior cingulate cortex; MeFG, medial frontal gyrus; CCRA, Chinese Compound Remote Association; CRRA, Chinese Radical Remote Association; CC, Chinese Compound Remote Associates Test-Correct; CU, Chinese Compound Remote Associates Test-Unsolved; RC, Chinese Radical Remote Associates Test-Correct; RU, Chinese Radical Remote Associates Test-Unsolved.

The contrast between the CC and CU suggests that the caudate, posterior cingulate cortex, postcentral gyrus, and medial frontal gyri were all activated. Similarly, the contrast between the RC and RU showed that only the caudate was activated. The results are presented in Table 2 and Figure 1.

**Table 2:** Significant brain regions for different types of remote associations.

**Side**	**Region**	**BA**	**Voxels**	**MNI Coordinates (mm)**			**Peak**
				**x**	**y**	**z**	***Z*** **-score**
**Chinese Compound Remote Association (Correct vs. Unsolved CCRAT)**							
R	Caudate	-	38	12	14	7	5.97
L	Postcentral gyrus	5	225	0	−46	70	5.36
R	Posterior cingulate cortex	31	45	6	−43	31	4.96
L	Medial frontal gyrus	10	21	0	68	19	4.85
**Chinese Radical Remote Association (Correct vs. Unsolved CRRAT)**							
R	Caudate	-	56	12	14	4	6.55

*CCRAT, Chinese Compound Remote Associates Test; CRRAT, Chinese Radical Remote Associates Test*.

#### Difference Among Remote Associations

**Figure 2:**
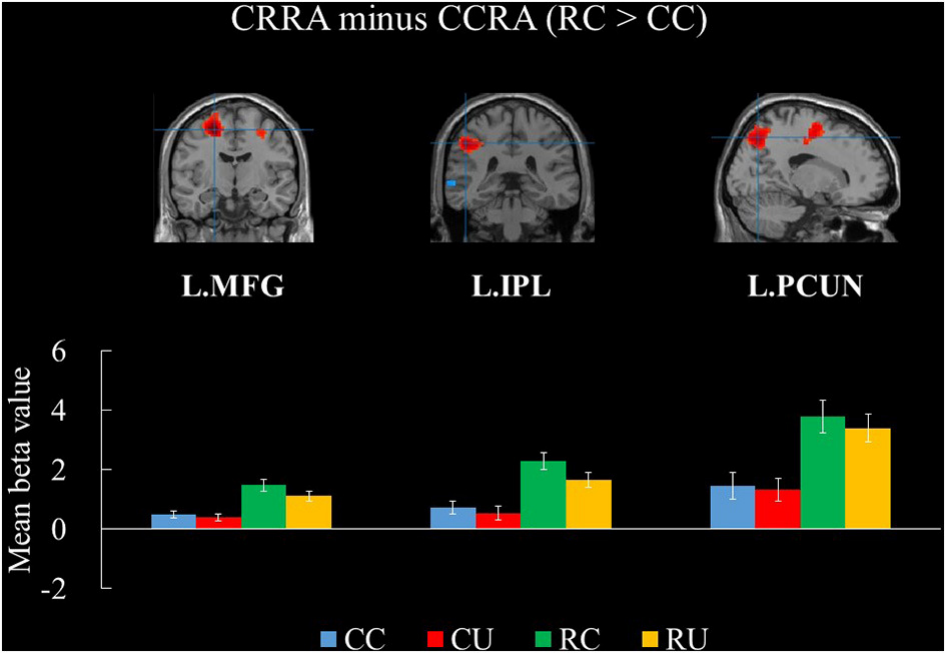
Brain regions for the contrast between Chinese Compound and Radical Remote Associations. The bars show the mean beta values of the peak voxels for each of the four conditions. Error bars represent standard error of the mean. L, left; MFG, middle frontal gyrus; IPL, inferior parietal lobule; PCUN, precuneus; CCRA, Chinese Compound Remote Association; CRRA, Chinese Radical Remote Association; CC, Chinese Compound Remote Associates Test-Correct; CU, Chinese Compound Remote Associate Test-Unsolved; RC, Chinese Radical Remote Associate Test-Corrected; RU, Chinese Radical Remote Associate Test-Unsolved.

This study revealed the difference between Chinese compound remote association and Chinese radical remote association. Chinese compound remote association minus Chinese radical remote association showed no significant activation in the brain regions. On the contrary, Chinese radical remote association minus Chinese compound remote association showed that the middle frontal gyrus, inferior parietal lobe, and precuneus were significantly activated, as shown in Table 3 and Figure 2.

**Table 3:** Significant brain regions for the difference among remote associations.

**Side**	**Region**	**BA**	**Voxels**	**MNI Coordinates (mm)**			**Peak**
				**x**	**y**	**z**	***Z*** **-score**
**CRRA minus CCRA (Correct CRRAT vs. Correct CCRAT)**							
L	Middle frontal gyrus	6	54	−24	−10	55	5.32
L	Inferior parietal lobule	40	37	−51	−34	43	4.76
L	Precuneus	7	33	−18	−70	49	4.71

*CCRAT, Chinese Compound Remote Associates Test; CRRAT, Chinese Radical Remote Associates Test; CCRA, Chinese Compound Remote Association; CRRA, Chinese Radical Remote Association*.

## Discussion

This research explored the differences in brain activities from the neural perspective to differentiate verbal and visual-spatial associations, distinguishing the brain mechanisms of different types of remote associations. These results are partially consistent with the hypothesis that Chinese compound remote association involves the activation of the brain regions of the caudate, posterior cingulate cortex, post-central gyrus, and medial frontal gyrus. The Chinese radical remote association led to a significant activation of the caudate alone. Chinese radical remote association minus Chinese compound remote association showed that the middle frontal gyrus, inferior parietal lobule, and precuneus were significantly activated. The results showed that the brain mechanism of Chinese radical remote association that involves visual-spatial abilities is different from that of Chinese compound remote association, which features verbal association. This is one of the first studies to present the differences in brain activity between verbal and visual-spatial remote associations.

The contrast between CC and CU reflects how one forms a remote association based on Chinese characters. The research results showed significant activation of the caudate, posterior cingulate cortex, postcentral gyrus, and medial frontal gyrus, indicating that these regions may all involve verbal remote association. The caudate is the center of language control (Crinion et al., 2006), including verbal fluency and transformation (Villablanca, 2010; Hsieh et al., 2017). The posterior cingulate cortex and medial frontal gyrus are important nodes in the default mode network (Gusnard and Raichle, 2001; Fox et al., 2005), which are positively correlated with divergent thinking (Ellamil et al., 2012; Gonen-Yaacovi et al., 2013; Beaty et al., 2015). In addition, the posterior central gyrus is involved in the mental representations of novel information processing (Huang et al., 2015). To conclude, whether one can form Chinese compound remote association lies in effective control and transformation over the verbal and mental representations and production of all possible novel ideas in a fluent manner. This finding is consistent with that of behavioral research (Wu, 2019), highlighting the significance of verbal association and divergent thinking in the CCRAT problem-solving process.

In terms of Chinese radical remote association, the contrast between RC and RU reveals how one forms remote associations based on Chinese radicals. The caudate was the only brain region that was significantly activated during the test. As mentioned above, the caudate involves verbal fluency and transformation (Hsieh et al., 2017), indicating that whether one can answer the CRRAT questions correctly is also associated with his/her verbal associative ability. Meanwhile, correct answers to both CRRAT questions and unsolved ones, which are two conditions, involve visual-spatial imagery abilities, for respondents to consider the relative position of stimulus Chinese radicals and target ones. Therefore, the contrast between these two conditions did not show brain regions associated with visual-spatial abilities. Therefore, both Chinese compound remote association and Chinese radical remote association involve verbal association; thus, the contrast between the two may reveal the brain mechanism when visual-spatial association is formed.

In particular, the caudate was significantly activated in both Chinese radical remote association and Chinese compound remote association. This finding shows that the caudate involves common components of verbal remote association. The caudate plays a vital role in monitoring and controlling language (Crinion et al., 2006; Becker et al., 2020). Previous studies have found that the caudate was positively associated with original idea generation (Jauk et al., 2015), creative writing (Lotze et al., 2014), and verbal divergent thinking (Takeuchi et al., 2010). The results of this study further reveal the function of the caudate in verbal remote association.

The RC minus CC revealed brain regions that involve visual-spatial association. The results showed that the middle frontal gyrus, inferior parietal lobe, and precuneus were significantly activated. These regions are associated with creative cognition (Ellamil et al., 2012), insight (Cranford and Moss, 2012), and representation changes (Wu et al., 2019). The inferior parietal lobe is associated with visual creativity (Ellamil et al., 2012; Huang et al., 2013; Pidgeon et al., 2016), which validates the key role of visual-spatial association in CRRAT. In addition, the brain regions of the middle frontal gyrus and precuneus are associated with insight-problem solving (Cranford and Moss, 2012; Wu et al., 2019), which corresponds to the findings of behavioral research. In comparison with CCRAT, CRRAT has a higher correlation with insight-problem solving (Wu, 2019). The above findings reveal the brain mechanism when visual-spatial association is formed, showing differences in the question-answer processes of CCRAT and CRRAT from a physiological perspective.

There are some limitations to the implementation of this study. First, this study assumed that CRRAT involves visual-spatial association based on the previous research finding that CRRAT is positively associated with visual divergent thinking (Wu, 2019), but it still needs to be confirmed using other criterion tasks whether visual-spatial imagery ability is the ability involved in visual divergent thinking and visual-spatial association, such as Raven's Progressive Matrices and Torrance Tests of Creative Thinking (Figural Version A). In addition, this research adopted fMRI to analyze the brain regions responsible for verbal and visual-spatial remote associations, but the verification of the CCRAT and CRRAT problem-solving sequences is still insufficient. In this respect, subsequent research is advised to refer to the research paradigm by Beeman et al. (2004) and compare and contrast the similarity and difference in time series between verbal and visual-spatial remote associations from the perspective of temporal resolution, such as electroencephalography (EEG). Finally, the Visual Remote Associates Test (Toivainen et al., 2019) uses pictures as stimuli. Further research is required on the difference in the visual-spatial association between the Visual Remote Associates Test and the CRRAT.

In conclusion, this study adopted fMRI to examine brain activity when verbal and visual-spatial associations are formed, and the corresponding brain regions are involved. The brain regions of the medial frontal gyrus, inferior parietal lobule, and precuneus are associated with different cognitive functions, such as ideation (Bendetowicz et al., 2017, 2018), visual processing (Ellamil et al., 2012; Wu C.-L. et al., 2016), and representation changes (Wu et al., 2019), which indicates the similarities and differences in the problem-solving process of CCRAT and CRRAT, and reveals the differences in the brain mechanisms when verbal and visual-spatial associations are made. This research deepens the understanding of brain neural activity when remote associations are formed in different ways.

## Data Availability Statement

The raw data supporting the conclusions of this article will be made available by the authors, without undue reservation.

## Ethics Statement

The studies involving human participants were reviewed and approved by Institution Review Board of the National Taiwan Normal University. The patients/participants provided their written informed consent to participate in this study.

## Author Contributions

C-LW collected and analyzed the data, wrote the initial draft of the manuscript, and assisted in literature review and discussion. H-CC designed this study. All authors approved the final version of the paper.

## Conflict of Interest

The authors declare that the research was conducted in the absence of any commercial or financial relationships that could be construed as a potential conflict of interest.

## Publisher's Note

All claims expressed in this article are solely those of the authors and do not necessarily represent those of their affiliated organizations, or those of the publisher, the editors and the reviewers. Any product that may be evaluated in this article, or claim that may be made by its manufacturer, is not guaranteed or endorsed by the publisher.
